# The role of C1orf50 in breast cancer progression and prognosis

**DOI:** 10.1007/s12282-024-01653-8

**Published:** 2024-11-28

**Authors:** Yusuke Otani, Atsushi Tanaka, Masaki Maekawa, Tirso Peña, Anna Rogachevskaya, Teruhiko Ando, Takuto Itano, Haruyoshi Katayama, Eiji Nakata, Toshifumi Ozaki, Shinichi Toyooka, Hiroyoshi Doihara, Michael H. Roehrl, Atsushi Fujimura

**Affiliations:** 1 Department of Pathology, Beth Israel Deaconess Medical Center, Boston, MA USA; 2 Harvard Medical School, Boston, MA USA; 3 Department of Orthopedic Surgery, Okayama University Graduate School of Medicine, Dentistry and Pharmaceutical Sciences, 2-5-1 Shikata-Cho, Kita-Ku, Okayama, 700-8558 Japan; 4 Department of General Thoracic Surgery and Breast and Endocrinological Surgery, Okayama University Graduate School of Medicine, Dentistry and Pharmaceutical Sciences, 2-5-1 Shikata-Cho, Kita-Ku, Okayama, 700-8558 Japan; 5 Department of General Surgery, Kawasaki Medical School General Medical Center, 2-6-1 Nakasange, Kita-Ku, Okayama, 700-8505 Japan; 6 Department of Cellular Physiology, Okayama University Graduate School of Medicine, Dentistry and Pharmaceutical Sciences, 2-5-1 Shikata-Cho, Kita-Ku, Okayama, 700-8558 Japan; 7 Neutron Therapy Research Center, Okayama University, 2-5-1 Shikata-Cho, Kita-Ku, Okayama, 700-8558 Japan

**Keywords:** C1orf50, Luminal A breast cancer, Cell cycle, Immune evasion, YAP/TAZ

## Abstract

Although the prognosis of breast cancer has significantly improved compared to other types of cancer, there are still some patients who expire due to recurrence or metastasis. Therefore, it is necessary to develop a method to identify patients with poor prognosis at the early stages of cancer. In the process of discovering new prognostic markers from genes of unknown function, we found that the expression of C1orf50 determines the prognosis of breast cancer patients, especially for those with Luminal A breast cancer. This study aims to elucidate the molecular role of C1orf50 in breast cancer progression. Bioinformatic analyses of the breast cancer dataset of TCGA, and in vitro analyses, reveal the molecular pathways influenced by C1orf50 expression. C1orf50 knockdown suppressed the cell cycle of breast cancer cells and weakened their ability to maintain the undifferentiated state and self-renewal capacity. Interestingly, upregulation of C1orf50 increased sensitivity to CDK4/6 inhibition. In addition, C1orf50 was found to be more abundant in breast cancer cells than in normal breast epithelium, suggesting C1orf50’s involvement in breast cancer pathogenesis. Furthermore, the mRNA expression level of C1orf50 was positively correlated with the expression of PD-L1 and its related factors. These results suggest that C1orf50 promotes breast cancer progression through cell cycle upregulation, maintenance of cancer stemness, and immune evasion mechanisms. Our study uncovers the biological functions of C1orf50 in Luminal breast cancer progression, a finding not previously reported in any type of cancer.

**Supplementary Information:**

The online version contains supplementary material available at 10.1007/s12282-024-01653-8.

## Introduction

Breast cancer is the most common cancer in women, and one of the first cancers for which pathological classification based on molecular phenotype was introduced [1, 2]. The molecular characteristics of each subtype have been widely and deeply understood, but the heterogeneity of the subtypes is not commonly reviewed [3, 4]. Defined as a subtype of hormone receptor positive cancer, estrogen receptor (ER)-positive breast cancer accounts for approximately 70 percent of all breast cancers [5, 6]. Selective estrogen receptor modulators (SERMs) and aromatase inhibitors have both improved prognosis in patients with ER positive breast cancer [7, 8]. However, it is known that approximately 20% of patients with ER-positive breast cancer have a poor prognosis and develop metastasis or recurrence even after hormone therapy [1, 9]. Various attempts have been made to improve the prognosis of these patients, including extending the duration of postoperative hormone therapy from 5 to 10 years and adding postoperative chemotherapy [10, 11]. Studies have proposed increasingly popular methods in risk classification via multi-gene expression assays as novel predictors for poor prognostic factors in hormone receptor-positive and human epidermal growth factor receptor 2 (HER2)-negative breast cancer patients [12, 13]. Yet, no single gene has been found to be the sole predictor for these patient groups nor for drug indication.

While searching for genes of unknown function that may be involved in cancer prognosis, Chromosome 1 Open Reading Frame 50 (C1orf50) exhibited a robust prognostic difference in melanoma patients according to the Human Protein Atlas. This observation led us to investigate whether C1orf50 also plays a role in breast cancer prognosis. Upon analyzing data from The Cancer Genome Atlas-Breast Invasive Carcinoma (TCGA-BRCA) dataset, we discovered that C1orf50 is strongly correlated with the prognosis of stage II Luminal A breast cancer, which is considered to have low biological malignancy among ER-positive breast cancers.

According to the National Center for Biotechnology Information database, two transcript variants have been identified in the human *C1orf50* gene. Variant 1 is protein-coding, and variant 2 is non-coding RNA. The protein encoded by variant 1 is named DUF2452 (domain of unknown function) and consists of 199 amino acids (approximately 26 kDa) in a hairpin-like structure with two helixes (predicted by AlphaFold). This amino acid sequence is conserved from *Caenorhabditis elegans*, *Drosophila melanogaster*, fish (e.g. *Danio rerio*), amphibians (e.g. *Xenopus tropicalis*), birds (e.g. *Taeniopygia guttata*) to mammals (e.g. *Mus musculus*). Still no orthologs have been identified in yeast, bacteria or plants, suggesting its specificity to certain higher-order organisms. Despite its evolutionary conservation, C1orf50 shows low tissue-specific expression in humans, and to date, no direct association with any disease has been reported.

In this study, we used biological, biochemical, and bioinformatic analyses to examine the role of C1orf50 in breast cancer progression. These analyses help to improve treatment outcomes by identifying new prognostic factors in breast cancer, while simultaneously identifying new potential drug targets for breast cancer therapies.

## Materials and methods

### Immunofluorescent analysis and confocal microscopy

Immunofluorescent analysis on the breast cancer tissue array was performed as previously described [14]. The breast cancer tissue array was purchased from TissueArray.com (catalog number: BRM961a). After deparaffinization with xylene, the section was incubated with HistoVT One (Nacalai Tesque) for antigen retrieval following the manufacturer’s instructions, blocked with 1% bovine serum albumin (BSA) (Sigma-Aldrich) in phosphate-buffered saline with 0.05% Triton X-100 (PBST) for 1 h at room temperature (~ 25 °C). The section was incubated with primary antibodies in BSA-PBST at 4 °C overnight. After washing with PBST three times, the section was then incubated with secondary antibodies in BSA-PBST at room temperature for 1 h and mounted with DAPI-Fluoromount-G (SouthernBiotech). The section was observed using a confocal microscope, LSM780 (Carl Zeiss AG). Details of the antibodies are described in Table S1.

### Cell cultures and treatments

Human breast cancer cell line MCF7 was obtained from the Japanese Collection of Research Bioresources (JCRB), and BT474, SK-BR-3, and MDA-MB-231 were from the American Type Culture Collection (ATCC). All cells were cultured at 37 °C containing 5% CO2 in high glucose Dulbecco’s modified Eagle’s medium (DMEM, Fujifilm-Wako) supplemented with 10% fetal bovine serum (FBS, Corning) and 1% penicillin/streptomycin/L-glutamine (Fujifilm-Wako). RNAi experiments were performed using siRNA, Lipofectamine RNAiMAX (Thermo Fisher Scientific), and Opti-MEM (Thermo Fisher Scientific). The following siRNAs were used in this study, listed as [Target gene/Source/Identifier]: [negative control/Thermo Fisher Scientific/4390844]; [human C1orf50/Thermo Fisher Scientific/s35534]; [human C1orf50/Thermo Fisher Scientific/s35535]; [human C1orf50/Thermo Fisher Scientific/s35536]. Lentiviral preparation and infection were performed as previously reported [15]. Briefly, 293FT cells were transfected with 10 μg viral backbone plasmids (pLKO.1-puro for shRNA and pTomo for overexpression), 7.5 μg psPAX2, and 2.5 μg pMD2.G using 60 μL TransIT-LT1 transfection reagent (TaKaRa Bio) and 1.5 mL Opti-MEM. The virus-containing medium was harvested and filtered with a polysulfone membrane. One milliliter of the medium was added dropwise to MCF7 or BT474 cells cultured on a 60 mm dish. The following target sequences of shRNA were used in this study: Human C1orf50 #1 [CTGCACCATGTAGCTTGTAAT]; Human C1orf50 #2 [GTCAGTCAGTTTCAGAGTATT]; Control [CCTAAGGTTAAGTCGCCCTCG]. Myc-tagged C1orf50 was synthesized by FASMAC (Kanagawa, Japan) and enzymatically cloned into a pTomo vector. The sphere-formation assay experiments were performed as previously reported [16].

### Immunoblotting analysis

Immunoblotting experiments were performed as previously described [17]. The cell lysate was prepared using cell lysis buffer (20 mM Tris–HCl (pH 7.5), 150 mM NaCl, 1 mM ethylenediaminetetraacetic acid, 1 mM ethylene glycol-bis(2-aminoethylether)-N,N,N’,N’-tetraacetic acid, 1% Triton X-100, cOmplete Protease Inhibitor Cocktail tablets (Roche), PhosSTOP phosphatase inhibitor cocktail tablets (Roche) and boiled in sodium dodecyl sulfate (SDS) sample buffer (50 mM Tris–HCl (pH 6.5), 100 mM dithiothreitol, 2% SDS, 1.5 mM bromophenol blue, 1.075 M glycerol). Equivalent amounts of each protein were loaded into acrylamide gel and transferred onto polyvinylidene fluoride (PVDF) membranes (Immobilon-P, 0.45 µm, Millipore). The membranes were then subjected to immunodetection using the antibodies listed below. The signals were detected using a ChemiDoc Touch Imaging System (Bio-Rad). Details of the antibodies are described in Table S2.

For the other materials and methods regarding bioinformatics analyses and statistics, see Doc. S1 and Table S1–2.

## Results

### Discovery of C1orf50 as a prognostic marker for Luminal A breast *cancer*

**Fig. 1:**
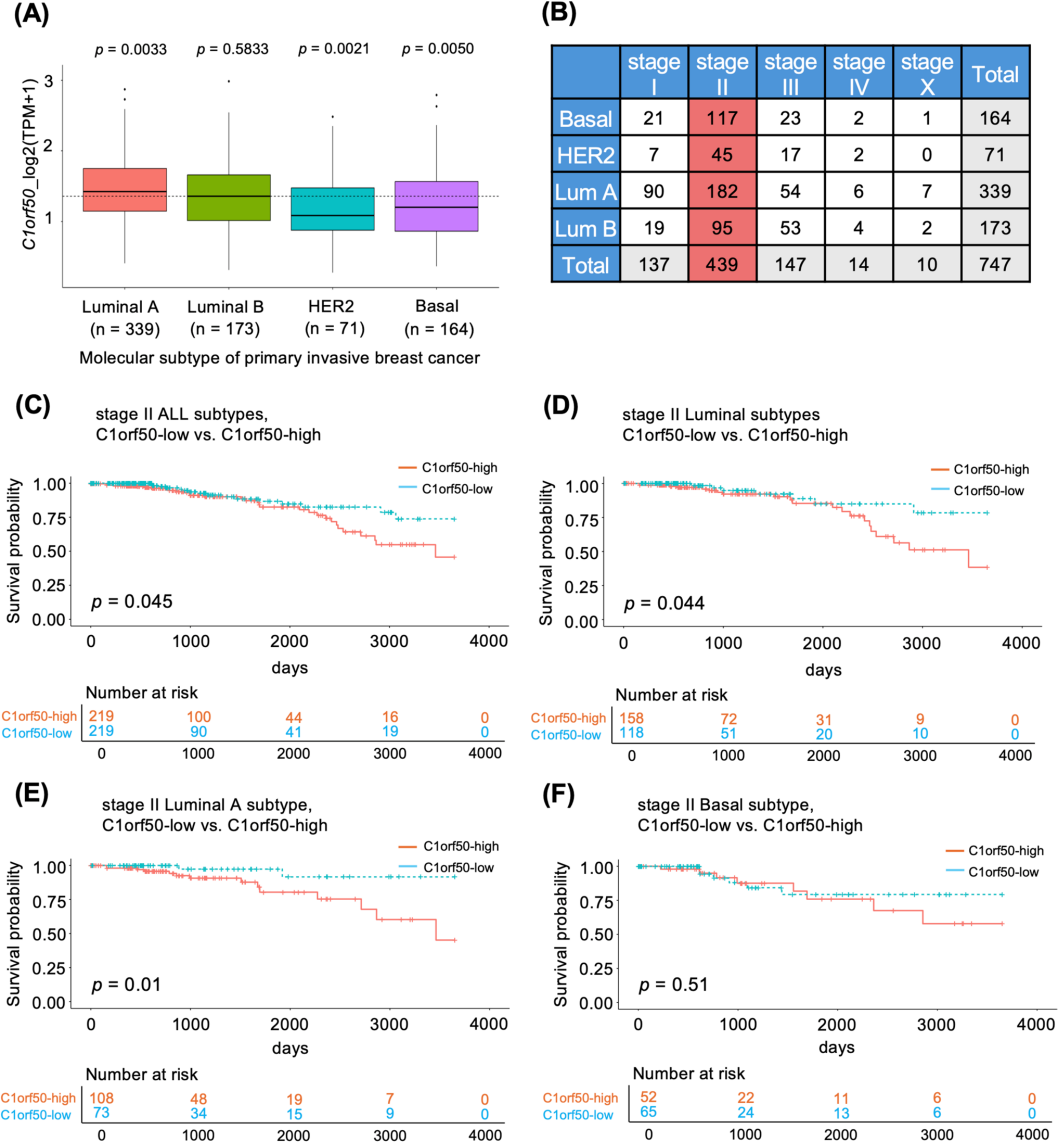
C1orf50 is a prognostic marker for Luminal A breast cancer. **A** The difference of the *C1orf50* expression value between subtypes in primary IDC (all stages). **B** Number of samples of stage I to IV primary IDC by subtype, in the TCGA database. Samples with unknown stages are labeled as stage X. **C–F** The Kaplan–Meier curves for 10-year overall survival in the C1orf50-high and C1orf50-low groups of stage II: all (**C**), Luminal (**D**), Luminal A (**E**), and Basal (**F**) subtype breast cancer patients

We investigated whether *C1orf50* mRNA expression plays a prognostic role using the TCGA-BRCA dataset, in which RNAseq data is accompanied by survival information. For the analysis, we included 747 primary tumor and invasive ductal carcinoma (IDC) cases in the TCGA-BRCA dataset. When comparing the expression levels of *C1orf50* by IDC subtype, it was found that Luminal A breast cancer showed significantly higher expression levels of *C1orf50* than non-Luminal A breast cancer (Fig. 1A). When examining the primary IDC breast cancer patient population registered in TCGA, it was found that there were many patients with stage II breast cancer (Fig. 1B). Since the prognosis for stage I breast cancer is widely known, this analysis focused primarily on patients with stage II breast cancer. Using the median of *C1orf50* mRNA expression values, we divided stage II breast cancer patients into C1orf50-high and C1orf50-low groups and performed survival analysis of stage II breast cancer patients, finding a significant (*p* = 0.045) difference in 10-year survival (Fig. 1C). In particular, a trend towards a greater divergence in the survival curve was observed for five years. In the analysis of stage II breast cancer patients by histological subtype, a significant difference in 10-year survival was observed in patients with Luminal-type breast cancer (*p* = 0.044), which is characterized by estrogen receptor expression (Fig. 1D). This difference was particularly significant (*p* = 0.01) in the group of patients with Luminal A breast cancer (Fig. 1E). There was no significant difference in 10-year survival rates among patients with triple-negative, HER2, and Luminal B stage II breast cancer (Fig. 1F and Fig. S1A, B). This data suggests that the expression level of *C1orf50* mRNA is a prognostic marker, especially in patients with stage II luminal A breast cancer. In addition, we investigated whether C1orf50 has different effects in premenopausal and postmenopausal breast cancer. In this study, we re-categorized women aged 50 years or older as postmenopausal. We found that C1orf50 may be a factor involved in survival in stage II breast cancer in postmenopausal patients (Fig. S1C-F).

**Fig. 2:**
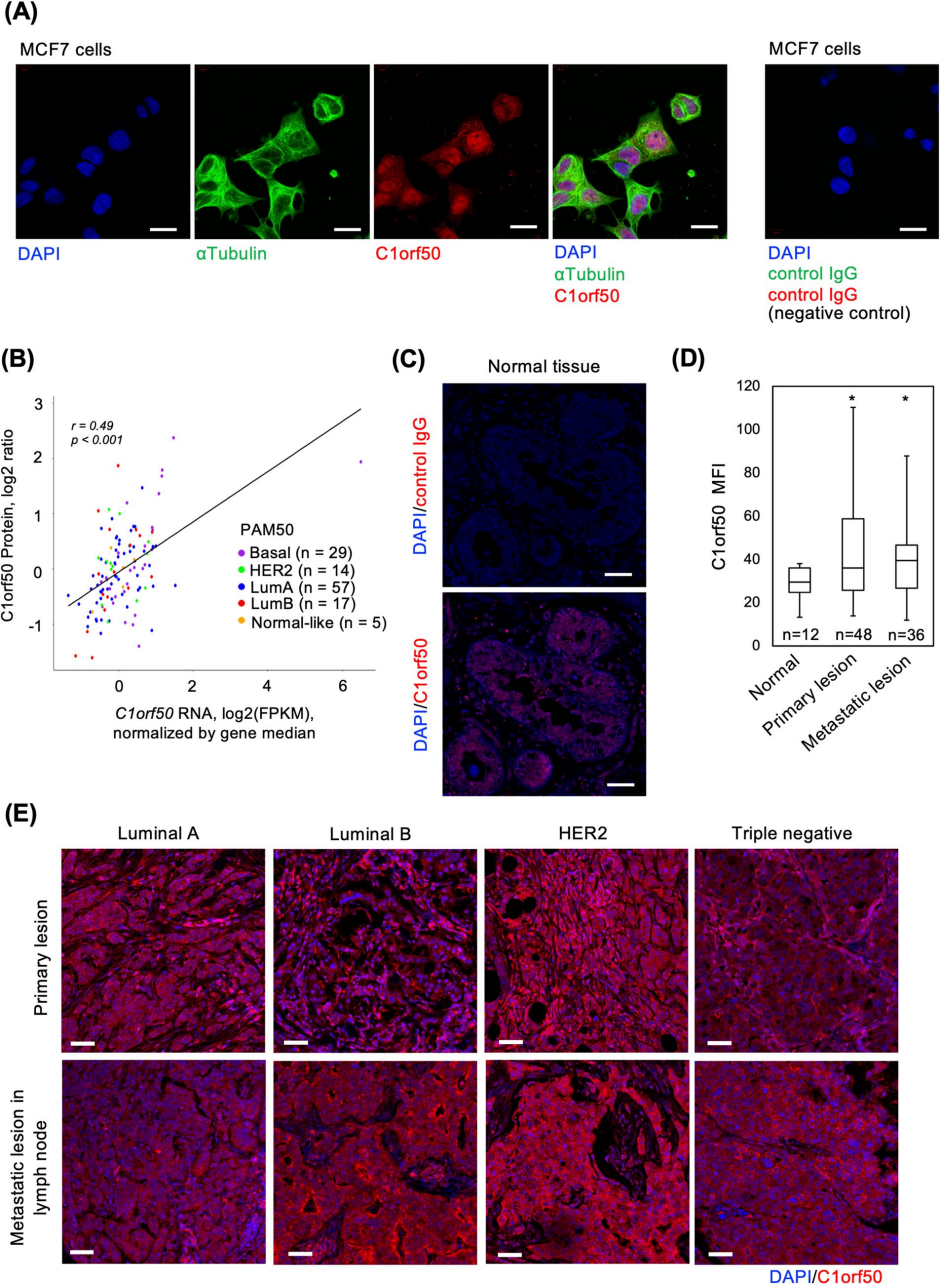
C1orf50 is abundantly expressed in breast cancer tissues. **A** Representative image of MCF7 cells immunostained with anti-αTubulin (green) and anti-C1orf50(red) antibodies. MCF7 cells immunostained with control IgG did not show any specific signals. Nuclei are stained with DAPI (blue): scale bar, 10 μm. **B** *C1orf50* mRNA and C1orf50 protein expression are positively correlated in the CPTAC dataset. Spearman’s rank correlation coefficient assesses the strength and direction of association between two ranked variables. **C** Representative image of normal breast tissue immunostained with control IgG (upper panel) or anti-C1orf50 antibody (lower panel) (red). Nuclei are stained with DAPI (blue): scale bar, 50 μm. **D** Using mean fluorescence intensity (MFI), C1orf50 expression is shown to be significantly higher in primary (*n* = *48*) and metastatic (*n* = *36*) breast cancer tissues compared to normal breast tissue (*n* = *12*). *: *p* < 0.05. Analysis was performed using one-way ANOVA with Tukey–Kramer’s multiple comparisons. **E** Representative immunostaining images using the anti-C1orf50 antibody in primary breast cancer lesion (top) and metastatic lesion in lymph node (bottom) with Luminal A, Luminal B, HER2, and triple-negative subtypes. Nuclei are stained with DAPI (blue): scale bar, 50 μm

Next, we determined whether C1orf50 is expressed at the protein level in breast cancer cells. In Luminal-type MCF7 cells, C1orf50 protein was detected both in the nucleus and cytoplasm at an endogenous level in immunofluorescent analysis (Fig. 2A). In MCF7 cells exogenously expressing myc-tagged C1orf50, the signal of endogenous C1orf50 overlapped with that of exogenous C1orf50 at the nucleus and cytoplasm (Fig. S2). Using a cohort of breast cancer patients from the Clinical Proteomic Tumor Analysis Consortium (CPTAC), we analyzed whether *C1orf50* mRNA and C1orf50 protein expression were correlated (*r* = *0.49, p* < *0.001),* and found a positive correlation (Fig. 2B). Furthermore, immunostaining with anti-C1orf50 antibody in tissue arrays composed of normal mammary tissues and breast cancer tissues showed that C1orf50 protein expression was low in normal mammary tissue (Fig. 2C). In contrast, the expression of C1orf50 protein was high in breast cancer tissues of all subtypes (Fig. 2D, E upper panels). Importantly, C1orf50 expression was found to be maintained at high levels not only in primary lesions but also in metastatic lesions of the lymph nodes (Fig. 2D, E lower panels). This data suggests that C1orf50 expression is upregulated in cancer cells compared to normal cells and that its expression is independent of the environment in which the cancer cells are located. In addition, the positive correlation between *C1orf50* mRNA and C1orf50 protein expression suggests that it is reasonable to evaluate C1orf50 protein as a prognostic marker in pathological specimens by immunostaining or other methods.

### Pathway analyses of C1orf50 in luminal A breast cancer

**Fig. 3:**
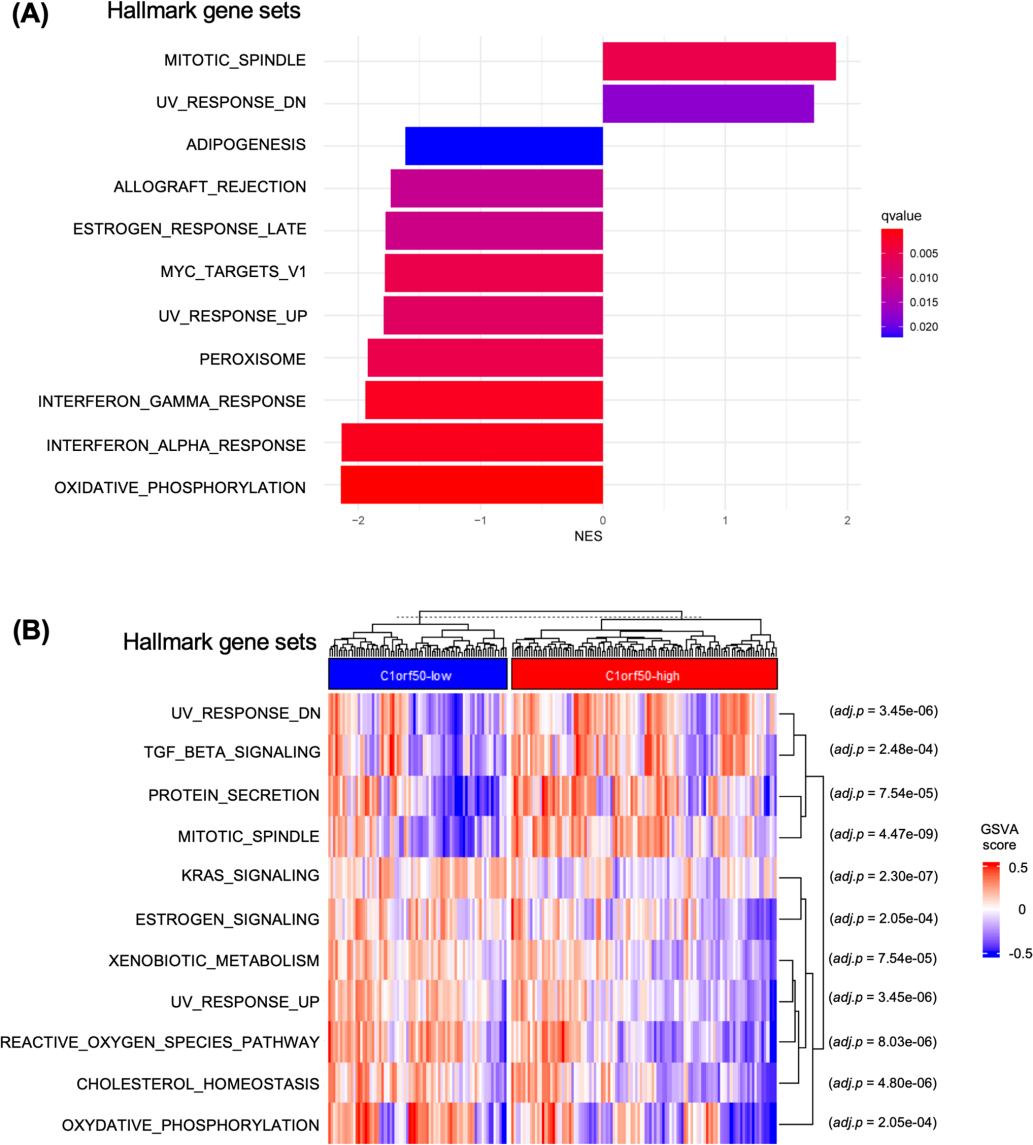
Pathway analyses show C1orf50 expression is associated with cell cycle activity, immunoreactive and estrogen-responsive gene groups in Luminal A breast cancer. **A** Gene set enrichment analysis (GSEA) of Hallmark gene sets, focusing on the C1orf50-high group of stage II Luminal A breast cancer. **B** Hallmark gene set terms with an adjusted p-value (*adj.p*) of less than 0.001 when comparing the C1orf50-high group (*n* = *109*) and the C1orf50-low group (*n* = *73*) in Gene Set Variation Analysis are shown

The data above shows that stage II breast cancer patients with high C1orf50 expression have a significantly worse prognosis (Fig. 1). The function of C1orf50 has not been previously reported, and its physiological and pathological roles and association with cancer biology remain completely unknown. To elucidate the molecular mechanisms of how C1orf50 promotes cancer progression, we performed pathway analysis focusing on *C1orf50* mRNA expression levels using the TCGA-BRCA dataset. First, we divided the TCGA data of stage II Luminal A breast cancer patients into C1orf50-high and C1orf50-low groups and performed Gene Set Enrichment Analysis (GSEA) using the Molecular Signatures Database (MSigDB) hallmark gene sets, and found a significant increase in the MITOTIC_SPINDLE gene set, which is related to the cell cycle. We also saw a decrease in the OXIDATIVE_PHOSPHORYLATION gene set, which is related to mitochondrial function, as well as a decrease in INTERFERON_ALPHA_RESPONSE, INTERFERON_GAMMA_RESPONSE, and ALLOGRAFT_REJECTION, which are related to immune response (Fig. 3A). Interestingly, we observed a reduction in the ESTROGEN_RESPONSE_LATE gene set: it has been reported that the value of this pathway correlates with estrogen reactivity [18], suggesting that a decrease in estrogen reactivity occurs in the C1orf50-high patient group. Furthermore, the Gene Set Variation Analysis (GSVA) confirmed a similar trend to the GSEA results, as well as a stronger association with transforming growth factor (TGF) beta signaling (*adjusted p* = 2.48e-04) in the C1orf50-high group (Fig. 3B). This data suggests that in stage II Luminal A breast cancer, patients with higher levels of C1orf50 expression are associated with an increased cell cycle activity. In comparison, lower levels of C1orf50 expression are associated with decreased expression of immunoreactive and estrogen-responsive gene groups.

Next, we performed the same analysis on the MsigDB C6 (Oncogenic signature) gene sets and found that the MEL18 gene signature and the BMI1 gene signature were decreased in the C1orf50-high group (Fig. S3A). MEL18 and BMI1 are Polycomb proteins involved in gene silencing [19]. In addition, since it has been reported that MEL18 deficiency leads to the reduction of estrogen receptors, which results in hormone-sensitive breast cancer cells acquiring the ability to grow in a hormone-independent manner [20], C1orf50 may have a vital role in hormone insensitivity in Luminal breast cancer patients. In the C6 gene set, several KRAS-related pathways are upregulated; previous studies have shown that increased expression of KRAS in the TCGA-BRCA dataset is associated with an increase in PD-L1 [21], suggesting that C1orf50 may indirectly contribute to immune reactivity or immune evasion. The Gene Ontology Biological Process (GOBP) gene sets showed a trend toward decreased pathways related to mitochondrial function, as well as reduced pathways related to immune response (Fig. S3B). Further analysis using the Kyoto Encyclopedia of Genes and Genomes (KEGG) gene sets, revealed decreased oxidative phosphorylation and decreased antigen processing and presentation (Fig. S3C). This data suggests that C1orf50 is widely implicated in cancer signaling, regulation of mitochondrial function, and immune evasion.

### Expression levels of C1orf50 determine cell cycle and response to CDK4/6 inhibitors of Luminal breast cancer

**Fig. 4:**
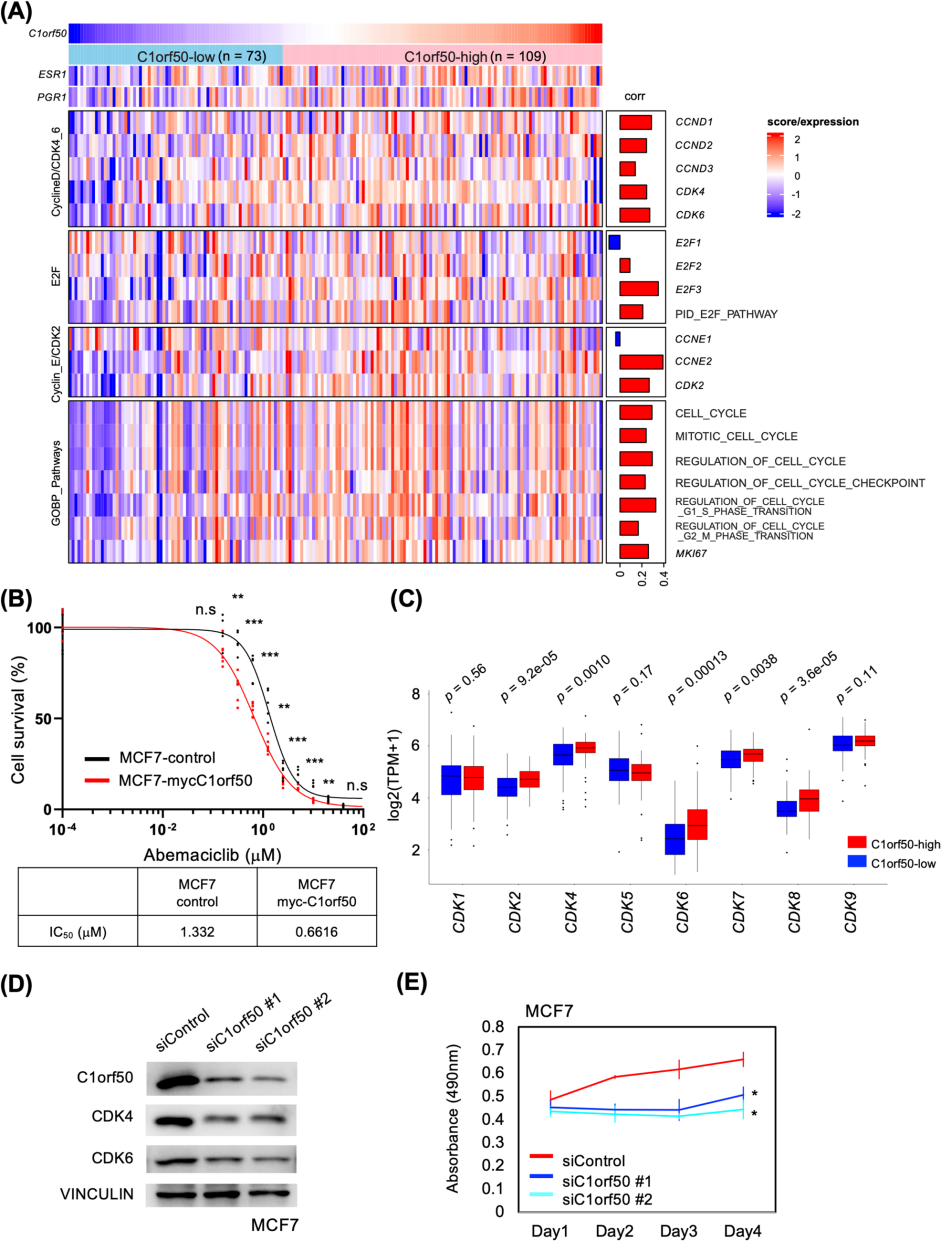
High expression levels of C1orf50 are pertinent to increased cell cycle signatures. **A** Heatmap of cell cycle-related genes and pathways focusing on *C1orf50* expression levels when comparing the C1orf50-high group (*n* = *109*) and the C1orf50-low group (*n* = *73*) of stage II Luminal A breast cancer in TCGA-BRCA dataset. **B** Cell survival analysis with serially diluted abemaciclib in MCF7-control and -myc C1orf50-transfected cells. IC_50_ values are shown in the table (*n* = *6*). **C** A box plot comparing RNA levels of *CDK1–9* in the C1orf50-low *(n* = *73)* and C1orf50-high groups *(n* = *109)*. *CDK3* is not shown because of the lack of expression data in the cohort. Comparisons between the two groups were performed using the Wilcoxon test. **D** Immunoblotting image of MCF7 cells transfected with siRNA. Both *C1orf50* #1 and #2 siRNA successfully attenuated the C1orf50 and CDK4/6 protein. VINCULIN serves as a loading control. **E** Growth curve of MCF7 cells transfected with siRNA. C1orf50 depletion significantly attenuated cell growth (*n* = *4*, error bars indicate mean ± SD). *p < 0.05. Analysis was performed using one-way ANOVA with Bonferroni’s multiple comparisons

Since the C1orf50-high group had enhanced cell cycle gene sets in both GSEA and GSVA, we analyzed the correlation between the expression level of C1orf50 and that of cell cycle-related factors in the TCGA-BRCA dataset. We found a positive correlation between the components of the cyclin D:CDK4/6 complex and the cyclin E:CDK2 complex (Fig. 4A). The cyclin D:CDK4/6 complex is a factor that promotes breast cancer progression [22, 23], and CDK4/6 inhibitors have recently been administered to patients with unresectable or recurrent Luminal breast cancer [24, 25]. The cyclin E:CDK2 complex has been reported to be involved in breast cancer progression [26, 27]. Interestingly, MCF7 cells exogenously transfected with C1orf50 showed increased sensitivity to abemaciclib, a CDK4/6 inhibitor drug (Fig. 4B), probably due to the increased dependency on CDK4/6 activity (Fig. S4A). We also observed a similar response to abemaciclib in BT474-myc C1orf50 cells (Fig. S4B). We compared the relationship between the expression levels of C1orf50 and eight CDKs in the dataset and found that the expression of CDK2, CDK4, CDK6, CDK7, and CDK8 was significantly higher in the C1orf50-high group (Fig. 4C). CDK7 forms a complex with cyclin H and MAT1 and acts as a CDK-activating kinase that phosphorylates and activates CDK2 and CDK4/6 [28]. It has been reported that when CDK8 expression is high in various carcinomas, especially breast cancer, a poor prognosis is found [29]. This data suggests that high levels of C1orf50 expression contribute to cell cycle acceleration.

To confirm that C1orf50 is associated with cell cycle progression, we transfected siRNAs against *C1orf50* mRNA into Luminal-type MCF7 cells. We also tested whether the proliferative capacity of the cells would be affected. Two unique siRNAs against *C1orf50* mRNA each attenuated the protein level of C1orf50 in MCF7 cells (Fig. 4D). This indicates that the anti-C1orf50 antibodies used in this study accurately recognize the C1orf50 protein. After siRNA transfection at 24, 48, 72, and 96 h, cell numbers were assessed using the MTS assay and showed that the proliferation of siC1orf50-transfected cells was significantly attenuated compared to siControl-transfected cells (Fig. 4E). We observed similar results in BT474 cells (Fig. S4C, D). This data indicates that the C1orf50 protein is indeed expressed in breast cancer cells and is imperative to cell cycle progression.

### C1orf50 promotes luminal breast cancer stemness properties

**Fig. 5:**
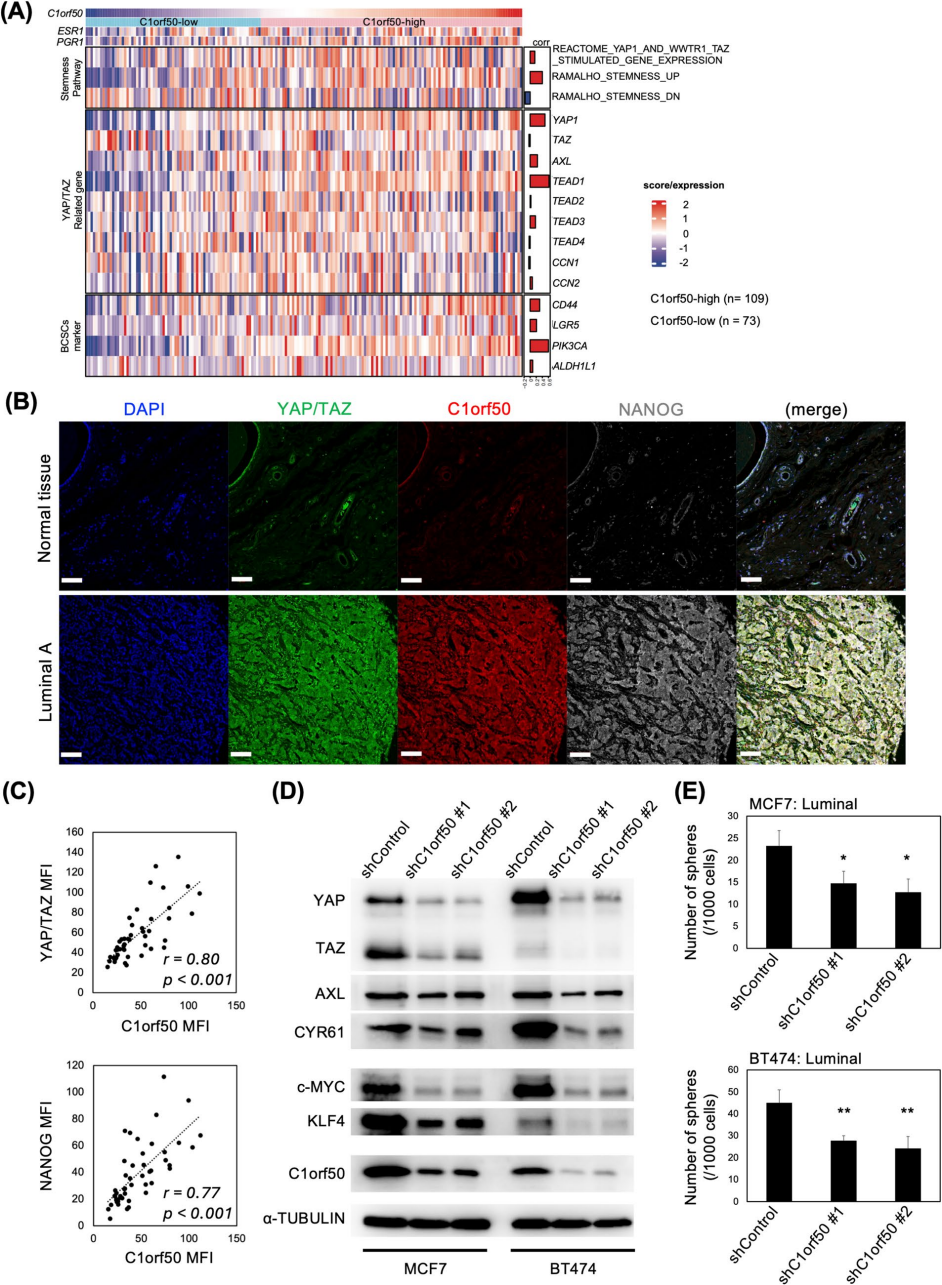
The expression of C1orf50 is essential to cancer stemness in Luminal breast cancer. **A** Heatmap of cancer stem cell-related genes and pathways focusing on *C1orf50* expression levels. The word “BCSCs” was used as an abbreviation for “Breast Cancer Stem Cells.” **B** Representative immunostaining images with anti-YAP/TAZ (green), anti-C1orf50 (red), and anti-NANOG (gray) antibodies in normal breast tissue and Luminal A breast cancer tissue. Nuclei are stained with DAPI (blue): scale bar, 100 μm. **C** C1orf50 MFI correlates with YAP/TAZ (*r* = *0.80, p* < *0.001*) and NANOG (*r* = *0.77, p* < *0.001)* MFI. Spearman’s rank correlation coefficient assesses the strength and direction of association between two ranked variables. **D** C1orf50 knockdown attenuated the protein levels of YAP and TAZ and their target genes AXL and CYR61. Loss of C1orf50 also decreases the expression levels of c-MYC and KLF4. Alpha tubulin serves as a loading control. **E** C1orf50 depletion leads to the loss of self-renewal capacity in Luminal breast cancer cells (*n* = *4*, error bars indicate mean ± SD). *p < 0.05, **p < 0.01, ***p < 0.001. Analysis was performed using one-way ANOVA with Bonferroni’s multiple comparisons

Cancer stem cell populations have been implicated in the poor prognosis of various cancers [30–32]. We confirmed that C1orf50 expression levels correlate with cancer stem cell-related signatures. Pathway analysis of the association between C1orf50 expression levels and cancer stemness showed that the stemness-related REACTOME_YAP1_AND_WWTR1_TAZ_STIMULATED_GENE_EXPRESS and RAMALHO_STEMNESS_UP scores were positively correlated with C1orf50 expression, and RAMALHO_STEMNESS_DN scores were negatively correlated with C1orf50 expression (Fig. 5A). The Hippo signal transducers, YAP/TAZ, are one of the most critical factors in the molecular mechanisms promoting cancer stem cells [15, 16, 33]. Many reports have shown that the expression level of YAP/TAZ defines cancer stemness [34]. Our data suggests that C1orf50 progresses breast cancer stemness through YAP/TAZ signaling.

Immunostaining with anti-YAP/TAZ, anti-C1orf50, and anti-NANOG antibodies in tissue arrays showed that C1orf50-high breast cancer cells express YAP/TAZ and NANOG at high levels in Luminal breast cancer cells (Fig. 5B). Having examined 48 human breast cancer samples, the C1orf50 mean fluorescence intensity (MFI) strongly correlates with both YAP/TAZ (*r* = *0.80, p* < *0.001*), and NANOG (*r* = *0.77, p* < *0.001)* MFI scores (Fig. 5C). We investigated the effect of C1orf50 on breast cancer cell stemness in vitro. First, we infected Luminal-type MCF7 and BT474 cell lines with lentiviruses expressing shRNA against *C1orf50* mRNA and performed Western blotting of cell extracts. We observed that C1orf50 protein deficiency results in decreased YAP/TAZ proteins. We confirmed that the expression levels of AXL and CYR61, target proteins of YAP/TAZ signaling, as well as the expression levels of c-MYC and KLF4, factors representing the cancer cell undifferentiated state, were similarly decreased (Fig. 5D). Since stemness is generally assessed by self-renewal capacity, we evaluated C1orf50-depleted breast cancer cells and confirmed that C1orf50 expression is imperative to the self-renewal capacity in breast cancer cells (Fig. 5E). This was not restricted to Luminal breast cancer cell lines, but also to other breast cancer molecular subtypes (Fig. S5). This suggests that C1orf50 is essential for the maintenance of breast cancer stemness.

### C1orf50 associates with immune evasion signatures in luminal breast cancer

We have shown that C1orf50 expression levels are particularly detrimental to prognosis in a subset of patients with Luminal A stage II breast cancer. The results of the Hallmark pathway analysis suggest that the patients with high C1orf50 expression may have suppressed immunity, but it remains unclear whether there is a patient population in Luminal breast cancer for whom immune checkpoint inhibitors, currently widely used in triple-negative breast cancer, are effective. Therefore, we performed in silico analysis to examine whether immune checkpoint inhibitors may be applicable in patients with high C1orf50 expression.

**Fig. 6:**
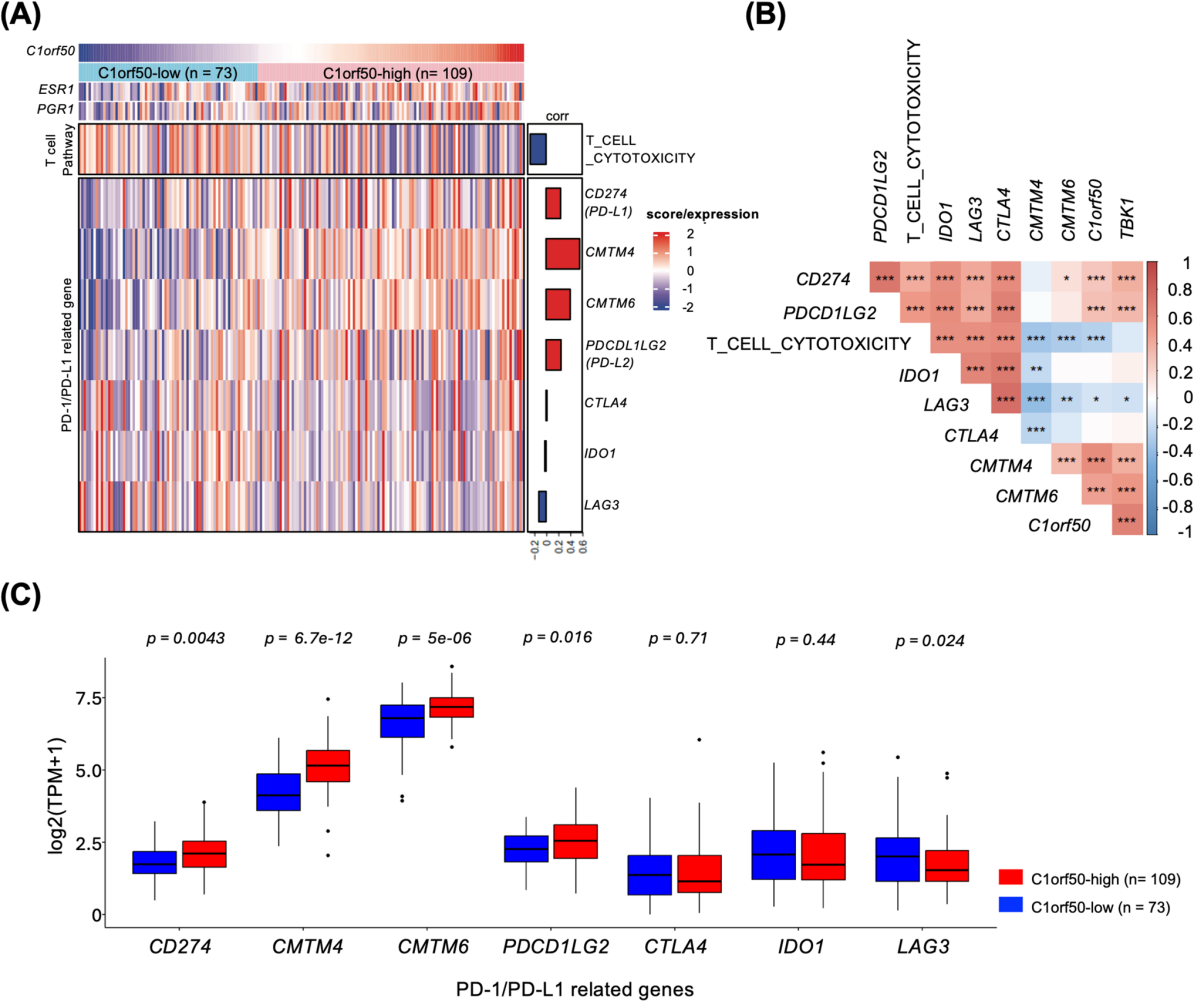
Correlation of C1orf50 expression with PD-1/PD-L1 related molecules in Luminal A stage II breast cancer. **A** Heatmap of PD-1/PD-L1 related genes and the GOBP_T_CELL_CYTOTOXICITY pathway compared across C1orf50 expression levels. The word “T_CELL_CYTOTOXICITY” in the heatmap was used as an abbreviation for “GOBP_T_CELL_MEDIATED_CYTOTOXICITY”. **B** Co-occurrence and mutual exclusiveness analysis of the immune-related genes and the GOBP_T_CELL_CYTOTOXICITY pathway. *p < 0.05, **p < 0.01, ***p < 0.001. Correlation tests were performed based on Pearson’s product-moment correlation coefficient. **C** A box plot comparing RNA levels of PD-1/PD-L1 related genes between the C1orf50-low and C1orf50-high groups. Comparisons between the two groups were performed using the Wilcoxon test

The Hallmark pathway analyses showed that immune response-related pathways were downregulated in the C1orf50-high group (Fig. 3A, B, Fig S3). We then found that C1orf50 expression negatively correlated with T-cell-mediated cytotoxicity (Fig. 6A). To further investigate the mechanisms behind these findings, we examined the mRNA expression levels of immunosuppressive molecules. As shown in Fig. 6A–C, the expression levels of PD-L1 (CD274) and PD-L2 (PDCD1LG2) were positively correlated with the expression level of C1orf50, and the expression levels of CMTM4 and CMTM6, regulators of PD-L1 [35], were also positively correlated with that of C1orf50. This data suggests that the expression level of C1orf50 may have a suppressive effect on immune checkpoint mechanisms regulated by PD-1/PD-L1. Therefore, the expression level of C1orf50 may be a useful marker when considering the application of PD-L1 inhibitors in the Luminal breast cancer patient population.

## Discussion

In this study, we investigated the role of the *C1orf50* gene, whose function was previously unknown in breast cancer progression, and confirmed that the prognosis is significantly worse in the group with high *C1orf50* mRNA expression by in silico analysis based on data from TCGA stage II breast cancer patients (Fig. 1B). Interestingly, this trend was more pronounced in patients with Luminal A breast cancer (Fig. 1D, E). In contrast, no significant correlation was observed between C1orf50 expression and prognosis in Basal-type (Fig. 1F), Luminal B (Supplementary Fig. 1A), or HER2-type breast cancer patients (Supplementary Fig. 1B). This suggests that the role of C1orf50 may be more specifically relevant to Luminal A subtype breast cancer. Moreover, we focused on stage II patients in this study, as the sample size for stage II was substantially more extensive compared to other stages, providing greater statistical power to detect significant associations. The clinicopathological significance of C1orf50 is that it may aid in improving the prognosis of Luminal A breast cancer patients according to C1orf50 expression levels. Current postoperative treatment of resectable hormone-positive HER2-negative breast cancer patients at risk of high recurrence is considered for CDK4/6 inhibitors in combination with endocrine therapy [24, 25]. Increased sensitivity to CDK4/6 inhibitors in breast cancer cell lines with C1orf50 high expression (Fig. 4B, Fig. S4B), and given that patients with high levels of *C1orf50* expression have a worse prognosis (Fig. 1D), aggressive administration of CDK4/6 inhibitors may be considered in Luminal breast cancer patients with high C1orf50 expression.

Since *C1orf50* mRNA has a coding sequence, it was predicted to be translated and expressed as a protein. Immunostaining and Western blot analysis confirmed that it can indeed be detected at the protein level in breast cancer tissues and cell lines (Figs. 2, 4D, 5B). To elucidate the molecular mechanism by which *C1orf50* mRNA expression affects Luminal breast cancer progression, we performed pathway analyses and found that the expression level of *C1orf50* mRNA is highly correlated with cell cycle and cancer stemness-related factors (Figs. 3, 4, 5). In breast cancer cell lines in which C1orf50 expression was knocked down by RNAi, cell proliferation was suppressed, and cancer stemness was significantly attenuated (Figs. 4 and 5). Importantly, in immunostaining analysis of normal breast tissues and breast cancer tissues, the latter tended to show higher C1orf50 staining (Fig. 2). This data shows that C1orf50 expression promotes breast carcinogenesis and progression, proving that the molecular mechanisms associated with C1orf50 are worthy of investigation as future drug targets.

A limitation of this study is that the details of the molecular mechanisms by which C1orf50 affects the cell cycle and immune checkpoint have yet to be discovered. To clarify this, future analyses focusing on the regulatory mechanism of the YAP/TAZ pathway by C1orf50 should be urgently performed. This is because the YAP/TAZ pathway has been shown to be a cell cycle driver in cancer cells [33], and there are reports that YAP/TAZ enhances PD-L1 expression and promotes immune evasion [36]. Furthermore, the TCGA-BRCA cohort used in this study had a limited number of HER2-type breast cancer cases, with only 71 patients registered. Additionally, the sample sizes for stage I tumors were small in both the Basal type (n = 7) and HER2 type (n = 21), which limits the statistical power for these subtypes. Therefore, further studies are warranted to explore the prognostic significance of C1orf50 in non-Luminal breast cancer subtypes, where differences may become more apparent with larger datasets.

Our study is important because it is the first worldwide report on the role of a functionally unknown gene, C1orf50, in cancer progression. It also provides clinically important insights in that further analysis of C1orf50 expression may change therapeutic intervention decisions in breast cancer.

## Conflict of interest

MHR is a member of the Scientific Advisory Board of Universal DX. This company had no influence on support, design, execution, data analysis, or other aspects of this study. The other authors have no conflict of interest.

## Ethics approval

Approval of the research protocol by an Institutional Reviewer Board. The human breast cancer tissue microarray was purchased from TissueArray.com (catalog number: BRM961a). The Ethics Committee of Okayama University concluded that individual review is not required for analyses using commercially available tissue arrays.

## Informed consent

Not applicable.

## Supplementary Information

Below is the link to the electronic supplementary material.

Supplementary file1 (PDF 1718 KB)

## Data Availability

Tha data supporting the findings of this study are available from the corresponding author upon reasonable request.

## Abbreviations

ER: Estrogen receptor
SERMs: Selective estrogen receptor modulators
HER2: Human epidermal growth factor receptor 2
C1orf50: Chromosome 1 open reading frame 50
TCGA: The cancer genome atlas
TCGA-BRCA: TCGA breast invasive carcinoma
CPTAC: Clinical proteomic tumor analysis consortium
MSigDB: The molecular signatures database
GSVA: Gene set variation analysis
IDC: Invasive ductal carcinoma
GSEA: Gene set enrichment analysis
YAP: Yes-associated protein
TAZ: Transcriptional co-activator with PDZ-binding motif
MFI: Mean fluorescence intensity
