# Supplementary material for: The role of C1orf50 in breast cancer progression and prognosis
Source: Breast Cancer. 2024 Nov 28;32(2):292–305. doi: 10.1007/s12282-024-01653-8 (PMC11842435; doi:10.1007/s12282-024-01653-8)
Supplement: Supplementary file 1 — Supplementary file1 (PDF 1718 KB) [file 12282_2024_1653_MOESM1_ESM.pdf]

Supporting Information

The Role of C1orf50 in Breast Cancer Progression and Prognosis

Yusuke Otani et al.

List of Supporting Information:

Doc. S1, 5 Supplementary figures, and 2 Supplementary tables

Doc. S1. Supplemental Materials and Methods

TCGA and CPTAC data download: All analyses using transcriptome data (count and transcripts per kilobase million [TPM] values) in The Cancer Genome Atlas (TCGA) were performed using R (version 4.3.2). The transcriptome data and clinical information of the TCGA Breast Invasive Carcinoma (TCGA-BRCA) dataset were retrieved using the R package, TCGAbiolinks (version 2.30.0). Among 1,231 samples, we selected 747 samples with metadata "infiltrating duct carcinoma, NOS" and "Primary solid Tumor" to reduce sample-wise heterogeneity. Based on the median of mRNA TPM values in stage II breast cancer, we divided 439 stage II breast cancer samples into two groups, named C1orf50-low (n = 219) and C1orf50-high (n = 219). Using log-transformed TPM values (Log2(TPM+1)), we conducted downstream analyses of the transcriptome data. One sample from the C1orf50-low group did not include survival data. The proteome data and RNA sequence data of the breast cancer samples analyzed were retrieved using the R package, CPTAC Biolinks (version 0.0.0.9000).

Differential expression analysis, gene set enrichment, and variation analysis: Using transcriptomics count data obtained from the TCGA dataset, we performed differential expression analysis using edgeR (version 4.0.7) and calculated fold-change values with q-values between specified groups. For C1orf50 comparison: in stage II Luminal A breast cancer patient samples, we divided tumor samples into two groups, C1orf50-low (n = 73) and C1orf50-high (n = 108) by the median TPM value of all stage II breast cancer subtypes. We used clusterProfiler (version 4.10.1) with a ranked gene list ordered by fold-change values to identify enriched pathways and biological processes. Using gene sets of the Molecular Signatures Database (MSigDB v2023.2.Hs), we conducted enrichment tests and calculated normalized enrichment scores (NES) for each term. For the functional characterization of each sample, we performed gene set variation analysis (GSVA) with MSigDB via the R GSVA package (version 1.50.1) and calculated the enrichment scores of the gene

sets. Differential GSVA score between the two groups was assessed using linear regression with the lmFit function from the limma package (version 3.58.1). A design matrix was created based on the group assignment, and statistical significance was determined using empirical Bayes moderation. Genes set with a p-value of < 0.001 were considered significantly differentially expressed.

Heatmap: The heatmaps were generated using the ComplexHeatmap package (version 2.18.0) in R. In Figure 3B, rows and columns were reordered after clustering based on Euclidean distance, grouping the samples into C1orf50-high and C1orf50-low clusters. In Figures 4A, 5A, and 6A, the heatmap was annotated on the right side to display correlation values calculated using the Spearman method. The correlation coefficients for each gene with respect to the RNA levels of C1orf50 were visualized with a barplot, where positive correlations were represented in red and negative correlations in blue.

MTS assay: MTS assay experiments were performed as previously described [17]. For the chemoresistance assay, 2,000 cells were seeded into each well of a 96-well plate in DMEM. The following day, serially diluted abemaciclib (Selleck) was added to each well. Dimethyl sulfoxide (DMSO) was used as a negative control. Cell viability was measured using the CellTiter 96 AQueous One Solution Cell Proliferation Assay Kit (Promega). To assess the effects of C1orf50 RNAi on the proliferation of BT474 cells, the cell viability was measured daily (over the 4-day period) using the same kit.

Statistical analyses: Comparisons of numerical values between two groups were performed using the Wilcoxon test. Comparisons of numerical values between three or more groups were performed using one-way ANOVA with Bonferroni's multiple comparisons. The comparison test details are written in each figure legend. A *p*-value less than 0.05 was considered to be statistically significant. The significance levels are defined as *: $p < 0.05$, **: $p < 0.01$, ***: $p < 0.001$. The Kaplan-Meier curves were visualized using the survminer R package (version 0.4.9). All survival analysis used the log-rank test to compare the two groups, C1orf50-high and C1orf50-low, in all survival analyses. The box plots were created using the ggpubr package (version 0.6.0) in R. In the heatmaps (Fig 4A, 5A, 6A), we used Spearman's rank correlation coefficient to assess the strength and direction of association between two ranked variables.

Supplemental figures and tables

Supplementary figure 1.

Kaplan-Meier curves for overall survival in (**A**) stage II Luminal B breast cancer patients, (**B**) stage II HER2 breast cancer patients, (**C**) stage II breast cancer patients (≥ 50 y/o), (D) stage II breast cancer patients (< 50 y/o), (E) stage II Luminal A breast cancer (≥ 50 y/o), (F) stage II Luminal A breast cancer (< 50 y/o).

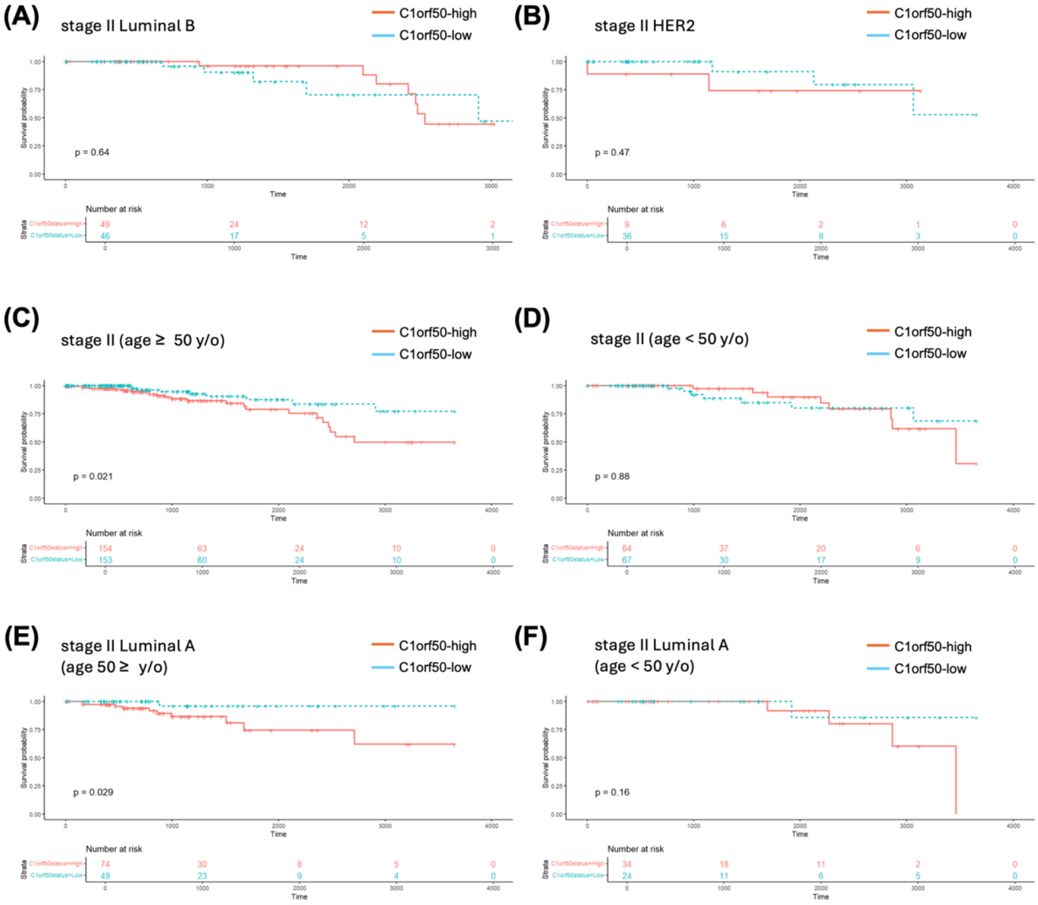

Supplementary figure 2.

Representative immunostaining images of MCF7-myc C1orf50 cells using anti-myc tag (green) and anti-C1orf50 (red) antibodies. Exogenous and endogenous C1orf50 signals overlapped, confirming that the C1orf50 primary antibody used in the study successfully recognizes C1orf50. Nuclei are stained with DAPI (blue). Scale bar, 10 µm.

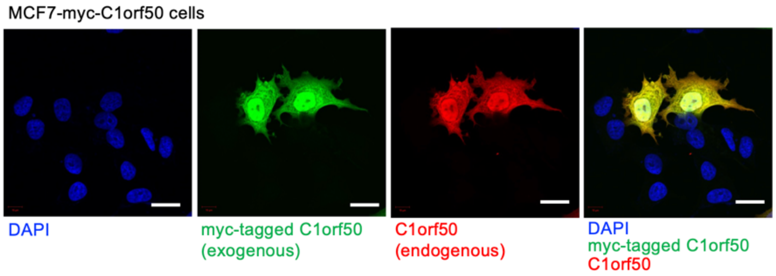

Supplementary figure 3.

Gene set terms with an adjusted p-value of less than 0.05 when comparing the C1orf50-high group (n = 109) and the C1orf50-low group (n = 73) in GSEA are shown. (**A**) C6 (Oncogenic signature) gene sets, (**B**) Gene Ontology Biological Process (GOBP) gene sets, (**C**) Kyoto Encyclopedia of Genes and Genomes (KEGG) gene sets.

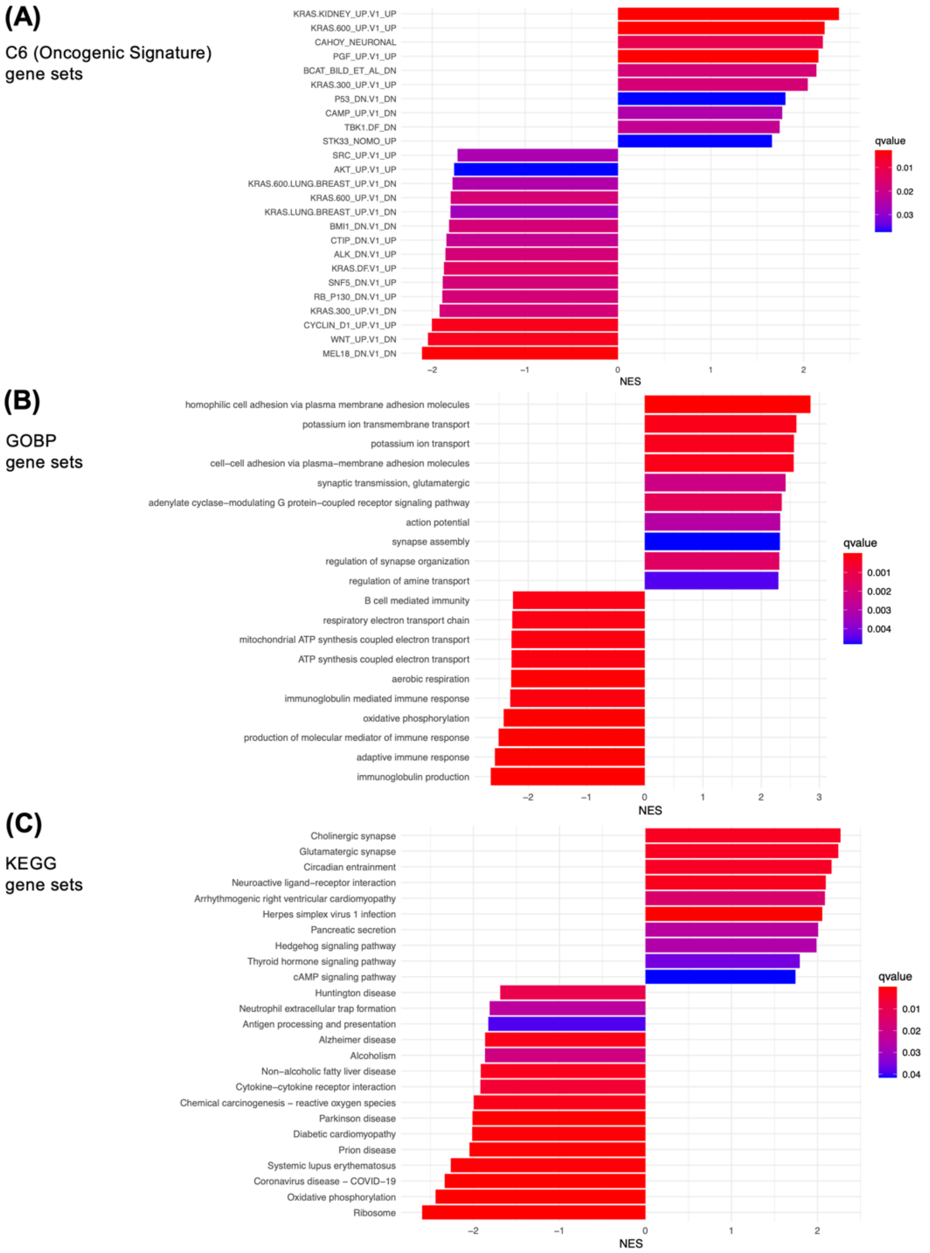

Supplementary figure 4.

C1orf50 expression levels are pertinent to breast cancer cell cycle signatures, related to Fig. 4. (**A**) Myc-tagged C1orf50 expression induces CDK4 and CDK6 in MCF7 cells. (**B**) Cell survival analysis with serially diluted abemaciclib in BT474-control and -myc C1orf50-transfected cells. IC_{50} values are shown in the table (*n = 6*). (**C**) Immunoblotting image of BT474 cells transfected with siRNA. Both C1orf50 #1 and #2 siRNA attenuated the C1orf50 protein. Histone H3 serves as a loading control. (**D**) Growth curve of BT474 cells transfected with siRNA. C1orf50 depletion significantly attenuated cell growth (*n = 4*, error bars indicate mean ± SD). **: $p < 0.01$, ***: $p < 0.001$. Analysis was performed using one-way ANOVA with Bonferroni's multiple comparisons.

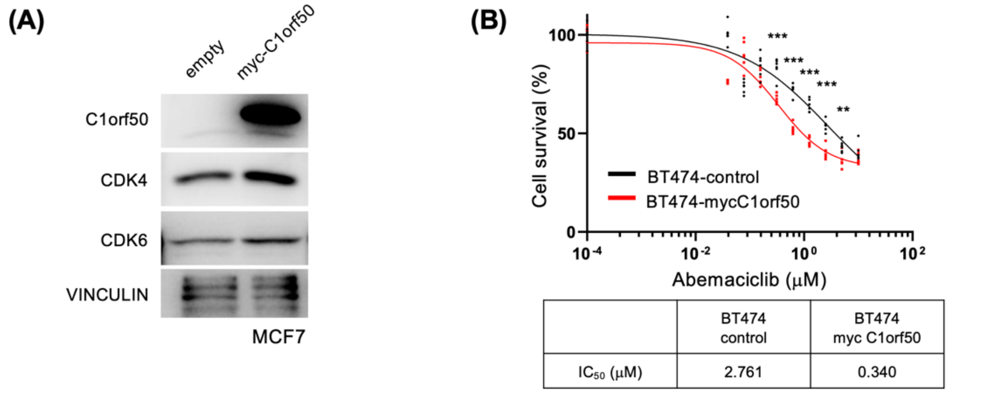

	BT474 control	BT474 myc C1orf50
IC_{50} (µM)	2.761	0.340

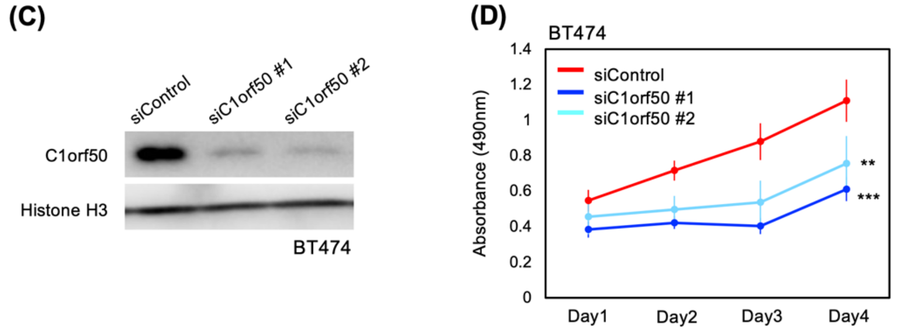

Supplementary figure 5.

C1orf50 depletion leads to the loss of self-renewal capacity in breast cancer cells (*n = 4*; error bars indicate mean ± SD). (**A**) SK-Br-3; HER2-subtype cell line, (**B**) MDA-MB-231; triple negative-subtype cell line. *: $p < 0.05$, **: $p < 0.01$, ***: $p < 0.001$. Analysis was performed using one-way ANOVA with Bonferroni's multiple comparisons.

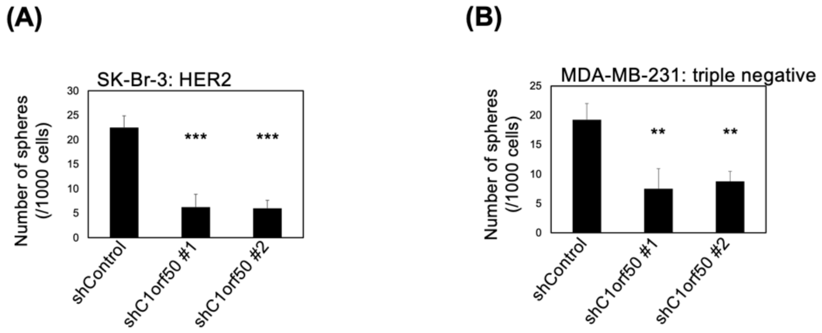

Supplementary table 1. Antibodies used for immunostaining.

Antigen	Source	Identifier	Dilution
C1orf50	Proteintech	20957-1-AP	1:100
YAP-TAZ	Santa Cruz Biotechnology	sc-101199	1:50
Nanog	R&D	AF1997	1:100
Donkey anti-mouse IgG Alexa Fluor Plus 488	Thermo Fisher Scientific	A32766	1:500
Donkey anti-rabbit IgG Alexa Fluor Plus 594	Thermo Fisher Scientific	A32754	1:500
Donkey anti-goat IgG Alexa Fluor Plus 647	Thermo Fisher Scientific	A32849	1:500

Supplementary table 2. Antibodies used for western blotting.

Antigen	Source	Identifier	Dilution
C1orf50	Proteintech	20957-1-AP	1:100
YAP-TAZ	Santa Cruz Biotechnology	sc-101199	1:50
Histone-H3	Proteintech	17168-1-AP	1:10000
Axl	Cell Signaling Technology	4939	1:2000
Cyr61	Cell Signaling Technology	39382	1:2000
c-Myc	Cell Signaling Technology	5605	1:2000
KLF4	Cell Signaling Technology	4038	1:2000
alpha-Tubulin	Proteintech	66031-1-Ig	1:20000
